# Molecular Mechanisms of Cardiac Remodeling and Regeneration in Physical Exercise

**DOI:** 10.3390/cells8101128

**Published:** 2019-09-23

**Authors:** Dominik Schüttler, Sebastian Clauss, Ludwig T. Weckbach, Stefan Brunner

**Affiliations:** 1 Department of Medicine I, University Hospital Munich, Campus Grosshadern and Innenstadt, Ludwig-Maximilians University Munich (LMU), 81377 Munich, Germany; Dominik.Schuettler@med.uni-muenchen.de (D.S.); Sebastian.Clauss@med.uni-muenchen.de (S.C.); Ludwig.Wekcbach@med.uni-muenchen.de (L.T.W.); 2 DZHK (German Centre for Cardiovascular Research), Partner Site Munich, Munich Heart Alliance (MHA), 80336 Munich, Germany; 3 Walter Brendel Centre of Experimental Medicine, Ludwig-Maximilians University Munich (LMU), 81377 Munich, Germany; 4 Institute of Cardiovascular Physiology and Pathophysiology, Biomedical Center, Ludwig-Maximilians-University Munich, 82152 Planegg-Martinsried, Germany

**Keywords:** physical exercise, cardiac cellular regeneration, microRNA (miR), Akt signaling, cardiomyocyte proliferation, cardiac hypertrophy, cardioprotection

## Abstract

Regular physical activity with aerobic and muscle-strengthening training protects against the occurrence and progression of cardiovascular disease and can improve cardiac function in heart failure patients. In the past decade significant advances have been made in identifying mechanisms of cardiomyocyte re-programming and renewal including an enhanced exercise-induced proliferational capacity of cardiomyocytes and its progenitor cells. Various intracellular mechanisms mediating these positive effects on cardiac function have been found in animal models of exercise and will be highlighted in this review. 1) activation of extracellular and intracellular signaling pathways including phosphatidylinositol 3 phosphate kinase (PI3K)/protein kinase B (AKT)/mammalian target of rapamycin (mTOR), EGFR/JNK/SP-1, nitric oxide (NO)-signaling, and extracellular vesicles; 2) gene expression modulation via microRNAs (miR), in particular via miR-17-3p and miR-222; and 3) modulation of cardiac cellular metabolism and mitochondrial adaption. Understanding the cellular mechanisms, which generate an exercise-induced cardioprotective cellular phenotype with physiological hypertrophy and enhanced proliferational capacity may give rise to novel therapeutic targets. These may open up innovative strategies to preserve cardiac function after myocardial injury as well as in aged cardiac tissue.

## 1. Introduction

Physical exercise has been shown to be protective against cardiovascular diseases (CVD), the leading cause of death worldwide [1]. Despite remarkable progress in medical, interventional, and surgical treatment options of CVD over the last years, prevention will be more and more vital for healthcare systems in an aging society. An alarming increase in the incidence of CVD-associated diseases such as insulin resistance, type II diabetes mellitus, and obesity demand altered lifestyle behaviors including dietary changes [2], cessation of smoking [3], and frequent physical exercise which all reduce risks for CVD clearly. The American Heart Association (AHA) therefore recommends at least 150 minutes of moderate-intensity aerobic activity or 75 minutes of vigorous aerobic activity or a combination of both per week as well as a moderate- to high-intensity muscle-strengthening activity on at least 2 days per week [4]. The beneficial effects on the cardiovascular system apply not only to young and healthy individuals [5] but also to patients with distinct cardiovascular risk factors or overt CVD and seem to decline after detraining [6,7]. Most importantly, exercise seems to be protective against myocardial ischemia-reperfusion injury [8].

Different acute and chronic changes in autonomic regulation, cardiac metabolism, signaling pathways, and protein expression in exercising hearts leading to cardiac growth and cellular reprogramming have been discovered over recent years. Especially, the beneficial effects of sports in heart failure and stable angina pectoris on patient outcomes, hospital admission, quality of life, and exercise capacity have been demonstrated [9,10,11]. However, the exact mechanisms of how physical activity delays the development of cardiovascular diseases remain unclear.

Physiologically, the heart can adapt to chronic exercise in order to meet the enhanced oxygen demand of the body, a process called ‘remodeling’. Exercise above three hours per week results in a significantly lower resting heart rate as well as significantly higher maximum oxygen uptake ($\dot{V}$O2) and left ventricular mass [12]. Aerobic training thus promotes physiological cardiac hypertrophy and can contribute to a preserved cardiac function. This has high clinical impact as training can counteract declined cardiac function to a certain extent in injured as well as in aging hearts.

Physiological hypertrophy is initiated via humoral factors and mechanical stress leading to changes in intracellular cardiac signaling to affect gene transcription, protein translation and modification, and metabolism [13]. These intracellular responses at a molecular level are different to those seen in pathological hypertrophy. In this context, exercise-modulated gene expression and cell signaling might protect the heart from further injuries and continuous maladaptive remodeling processes. Further understanding and identification of pathways responsible for physiologic exercise-induced adaption resulting in a cardioprotective phenotype with physiological hypertrophy and proliferation might help to identify triggers for physiologic/cardioprotective and pathologic/maladaptive remodeling that could potentially be used therapeutically to maintain cardiac function after ischemic or infectious injury of the heart as well as in aging hearts.

In the following review, we highlight these cellular mechanisms of cardiac remodeling in response to physical exercise with a focus on signaling pathways and microRNAs. 

## 2. Cardiac Cellular Changes in Exercise

### 2.1. Cellular Regeneration and Physiological and Pathological Hypertrophy

Injuries to the heart such as biochemical stress, toxins, infections, or ischemia require regeneration in order to maintain proper cardiac function. In contrast to most other cells, however, adult mammalian cardiomyocytes lose the ability to proliferate resulting in a low cellular turnover rate of 0.3 to 1% per year in the heart [14,15]. Although pioneering reports lately showed that adult mammalian cardiomyocyte proliferation could potentially be targeted via highly conserved signaling cascades such as peroxisome proliferator-activated receptor delta (PPARδ) agonist carbacyclin (induction of PPARδ/PDK1/protein kinase B (Akt) pathway) [16], extracellular matrix (ECM) protein agrin [17], or Hippo-Yap pathways [18], this very low cellular turnover is insufficient to achieve sufficient cardiac regeneration after cardiac injury.

Instead of regeneration with full restoration of organ function, hypertrophy, and fibrotic healing with incomplete functional recovery occur in the heart in response to injury. Regarding hypertrophy, one has to distinguish physiologic hypertrophy (proportional growth of length and width with proportional chamber enlargement) in response to exercise and pathologic concentric (relatively greater increase in width with disturbed contractile elements) as well as eccentric hypertrophy (relatively greater increase in length) in response to injury [19,20].

In this context sports have been shown to induce physiologic cardiac hypertrophy. In mouse models exercise induced inhibition of the transcription factor C/EBP beta and increased expression of CBP/p300-interacting protein with ED-rich carboxy-terminal domain-4 (CITED4) resulting in cardiac hypertrophy and proliferation, and contributed substantially to resistance against adverse cardiac remodeling and subsequent heart failure [21]. CITED4 has been demonstrated to mediate its effect on hypertrophy and recovery after ischemic injury due to its regulation of mammalian target of rapamycin (mTOR) signaling [22]. Cardiac cell proliferation, per se, does not seem to be necessary for exercise-induced cardiac growth but is highly required as a protective mechanism counteracting ischemia/reperfusion injury [23]. In addition to an enhanced proliferation and division of differentiated cardiomyocytes exercise such as swimming activates C-kit and Sca1 positive cardiac progenitor cells. These adult cardiac progenitor cells provide a certain potential of self-renewal and support myocardial regeneration [24]. This positive effect of sports on stem cell recruiting has been detected in the heart and the vascular system: Exercise-induced activation of cardiac and endothelial progenitor cells protects the heart, the coronary, and vascular system and attenuates the decline in arterial elasticity mediating positive effects on hypertension [25,26,27]. Administration of exogenous stem cells has been linked myocardial repair and regeneration in cardiac diseases and could be a potential therapeutic option in the future [28].

Nevertheless, even though these recent reports show that cardiomyocytes are at least to some degree capable of proliferation, adult hearts mainly respond to exercise and stress with an increase in cell size. Athletes’ hearts are consequently characterized by a benign increase in heart mass as an effect of regular training. This physiological transformation with increases in cardiac mitochondrial energy capacity has to be distinguished from pathological cardiac growth due to e.g., hypertension with diminished contractile function, ATP deficiency, and mitochondrial dysfunction [13,29,30]. Both physiological and pathological hypertrophy are associated with higher heart mass and size. Pathological hypertrophy, however, is associated with increased interstitial fibrosis, apoptosis, and loss of cardiomyocytes. It shows fetal gene expression, altered cell signaling, and a different metabolism with decreased fatty acid metabolism which results in cardiac dysfunction with increased risk of heart failure and sudden cardiac death compared to physiological cardiac hypertrophy in exercised hearts [19,20]. Activation of fetal genes is as mentioned above one of the prominent changes found in pathological cardiac hypertrophy. Alterations in gene expression patterns in hypertrophic hearts resemble patterns found during fetal cardiac development and involve regulations on transcriptional, posttranscriptional, and epigenetic level. This reactivation of a fetal gene program in failing hearts has been nicely reviewed by Dirkx et al. [31].

Physiological “benign” hypertrophy declines after long-term detraining with significant reduction in cavity size and normalization of wall thickness whereas pathological “malign” hypertrophy persists [32]. 

Reports of sudden cardiac deaths (SCD) among young athletes have gained pronounced attention in the media as well as in research over the past years and have initiated the debate whether there is a threshold between benign “healthy” hypertrophy and malignant “unhealthy” hypertrophy with pathological conditions due to regular high-intensity exercise. To date, there is no clear evidence that healthy athletes without an underlying cardiovascular disease or a genetic cardiomyopathy have an increased risk of SCD [33], although recent data reveals that the leading finding associated with SCD among athletes is actually a structurally normal heart (unexplained autopsy-negative sudden cardiac death) [34]. 

Nevertheless, there is strong evidence for beneficial effects of regular aerobic and muscle-strengthening activity despite the association with mild cardiac hypertrophy.

### 2.2. Animal Models of Exercise

To experimentally study exercise-induced cellular cardiac alterations and their effects on cardiovascular health, aging, and response to injury, various animal models of physical exercise have been developed over the years using zebrafish, rodents, and large animals (rabbits, dogs, pigs, goats, sheep, and horses). Exercise modalities mainly include swimming or treadmill and wheel running [35].

The zebrafish as an animal model has gained broad attention in the field of exercise and regeneration physiology of the heart in the past years [36,37]. Unlike mammals, adult zebrafish hearts are capable of proliferation in case of cardiac injury and therefore represent a valuable model to study cardioprotection, regeneration, and aging [38,39]. This plasticity of the zebrafish heart has been demonstrated in injured hearts [40,41] as well as in intensified swimming-trained hearts [36] and revealed useful insights in cardiac remodeling processes and their cellular basis.

Rodents, especially rats and mice, are the most frequently used species to investigate effects of sports on cardiovascular system due to several advantages: Short gestation periods, syngeneic strains, relatively low housing costs, and easily reproducible experiments [42]. Most importantly, however, exercise-induced cardiac hypertrophy in mice shows physiological cardiac responses similar to those seen in humans [43] which makes them a valuable pre-clinical model. Rodent exercise models therefore have been widely used to study effects on cardiac hypertrophy as well as regeneration and aging: Broad insights in aging processes including telomere shortening have been gathered in exercising mice and rats [44]. Physical activity has been highly useful to study exercise-induced cardiac hypertrophy and to distinguish it from its pathological form [35] as observed e.g., in mouse models of aortic constriction [45]. These models revealed alterations in intracellular signaling such as Akt/mTOR/S6K1/4EBP1 pathways [46,47,48]. In rodents, treadmill running is predominantly used as this modality allows to adjust different intensities including interval training as well as modulations in inclination, speed, and duration. These intensity-controlled treadmill workouts provide reproducible cardiac hypertrophy and increase heart weights by 12–29% and cardiomyocyte dimensions by 17–32% in mice [49]. Beneficial effects on cardiovascular function mediated by treadmill running with high intensity interval training (HIIT) or long-term aerobic exercise before myocardial infarction include for example significantly reduced infarct sizes as well as an increased induction of anti-apoptotic effects in cardiac cells [50,51].

In contrast, larger animals are rather infrequently used compared to rodent models to study exercise-induced effects on the heart due to higher costs and efforts in housing and experimental procedures. Nevertheless, especially pigs closely resemble human coronary and vascular anatomy, hemodynamic physiology, and electrophysiology [52]. Thus, pigs could be used to investigate exercise-induced cardiac effects. In this context, for example, improvements in myocardial contractile function and in collateral capacity after physical activity were detected in an ischemic porcine model [53].

### 2.3. Major Signaling Pathways in Exercise-Induced Cardiac Remodeling

**Figure 1:**
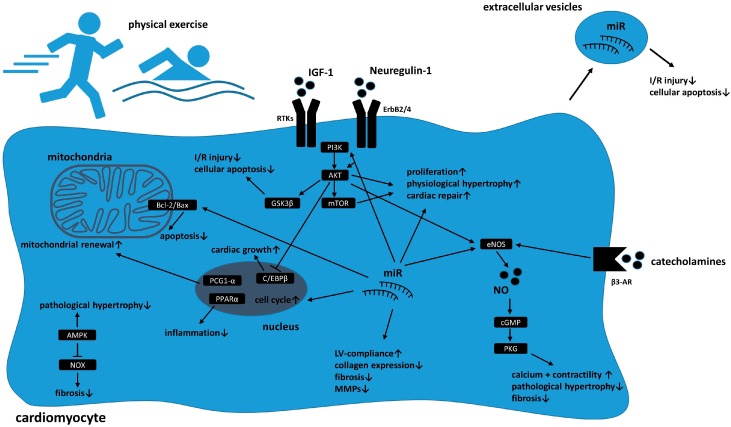
Schematic overview of cellular reprogramming in cardiomyocytes in response to physical exercise. Activation of receptor tyrosine kinases (RTKs) such as ErbB2/4 via growth factors (e.g., insulin-like growth factor 1 (IGF-1) or Neuregulin-1) enhance phosphatidylinositol 3 phosphate kinase (PI3K)/protein kinase B (Akt)/mammalian target of rapamycin (mTor)/ glycogen synthase kinase 3 beta (GSK3β) signaling which leads to proliferation, physiological hypertrophy, and cardiac repair mechanisms in response to injury. Beta3-adrenergic receptor (β3-AR) activation enhances endothelial nitric oxide synthase (eNOS) and subsequently intracellular nitric oxide (NO) levels which increases contractility and decreases fibrosis as well as pathological hypertrophy. Changes in miR expression influence intracellular signaling pathways (including Akt and eNOS), mediate apoptosis and cell cycle progression and influence cardiac compliance and fibrosis via alterations in collagen production and matrix metalloproteinase (MMP) expression. Sports induces mitochondrial renewal and decreases apoptosis via changes in B-cell lymphoma 2 (Bcl-2)/Bcl-2-associated X protein (Bax) ratio. Activation of adenosine monophosphate-activated protein kinase (AMPK) attenuates pathological hypertrophy and decrease profibrotic remodeling. Paracrine secretion of extracellular vesicles containing miR mediate I/R injury as well cellular apoptosis.

Over the past years different signaling pathways have been identified mediating cardioprotective cardiac growth and adaption as well as damage repair and attenuating cellular aging in response to physical exercise. Figure 1 provides an overview of cellular reprograming in cardiomyocytes in response to physical exercise.

#### 2.3.1. Akt-Signaling

The cellular key pathway in the regulation of physiological cardiac hypertrophy in response to exercise include phosphatidylinositol 3 phosphate kinase (PI3K) and Akt with their respective downstream signaling. These pathways are activated by extracellular growth factors such as insulin-like growth factor 1 (IGF-1) that has been linked to cardiac disease especially heart failure as it influences cardiac hypertrophy and contractile function [54]. This has been further demonstrated in rodents with heart failure where insulin-like growth factor-1 enhances ventricular hypertrophy and function [55] and suppresses apoptosis of cardiomyocytes [56]. Acute and chronic physical activity increase the local IGF-1 levels further confirming an important role in response to physical exercise but the precise role of IGF-1 is still not completely understood [57].

Mullen et al. identified PI3K and Akt signaling as crucial in the development of physiological (due to exercise) but not pathological (due to pressure overload) hypertrophy in mice [58]. These protective effects of enhanced PI3K signaling have been further demonstrated in dilated and hypertrophic cardiomyopathy with exercise and increase PI3K activity prolonging survival in a model of dilated cardiomyopathy up to 20% [59]. This implies that PI3K is essential for exercise-induced cardioprotection. Delivery of a constitutively active PI3K vector as a gene therapy can improve function of the failing heart in mice [60]. Akt acts as effector kinase downstream of PI3K and further activates mTOR signaling. Exercise training seems to be associated with activation of the Akt/mTOR pathway in contrast to pressure overload which is associated with its inactivation. This particular pathway may be one of the key regulators to distinguish between physiological and pathological cardiac hypertrophy. This cardiac growth is further mediated via downstream effectors of mTOR, including S6Kinase 1 and 4EBP1, as they play a crucial role in regulating protein biosynthesis, cell cycle progression, and hypertrophy [61,62].

Alterations of PI3K/Akt signaling is not only initiated via extracellular growth factors but also via changed intracellular microRNAs (miR) levels. miR-124 cluster among others suppress PI3K and are decreased in response to physical exercise leading to an enhanced activation of PI3K [63]. These changes mediated by miRNAs will be further elucidated in the next section.

Besides the mTOR pathway, Akt also mediates glycogen synthase kinase 3 beta (GSK3β) signaling. This key cellular pathway is involved in cellular apoptosis and alterations have been linked to various diseases including diabetes mellitus and carcinomas and was found to be altered in response to sports [64,65,66,67]. The role of GSK3 family in cardiac disease such as hypertrophy, aging, ischemic injury, or fibrogenesis has been shown in many rodent studies and has been comprehensively reviewed by Lal et al. [68]. Among this family: Akt; adenosine monophosphate-activated protein kinase (AMPK); beta3-adrenergic receptor (β3-AR); B-cell lymphoma 2 (Bcl-2); Bcl-2-associated X protein (Bax); CCAAT-enhancer-binding protein 1 (C/EBP1); cyclic guanosine monophosphate (cGMP); endothelial nitric oxide synthase (eNOS); GSK3β; IGF-1; ischemia/reperfusion (I/R); left ventricular (LV); miR; matrix metalloproteinase (MMP); mTOR; nitric oxide (NO); nicotinamide adenine dinucleotide phosphate (NADPH) oxidase (NOX); peroxisome proliferator-activated receptor gamma coactivator 1-alpha (PCG-1α); PI3K; PPARα; protein kinase G (PKG); receptor tyrosine kinase (RTK); and especially GSK3β seems to mediate positive effects in the context of ischemia/reperfusion injury which were found to be at least partly mediated via PI3K/Akt signaling [51].

#### 2.3.2. Neuregulin-1/ErbB-Signaling

The neuregulin-1/ErbB2/ErbB4 pathway is another key pathway which was found to be altered in response to physical exercise and is connected with Akt/PI3K signaling underlining its cellular significance. Activation of the tyrosine kinases ErbB2 and ErbB4 by neuregulin-1 in response to exercise activates PI3K/Akt signaling and protects ventricular myocytes against apoptosis as demonstrated in a myocardial infarction model in rats [69,70]. ErbB2 and its downstream cascades have been shown vital in promoting mammalian heart regeneration, cardiomyocyte dedifferentiation, and proliferation [71]. In the past years this pathway has thus gained broad interest as potential therapeutic target in failing and ischemic hearts.

Neuregulin-1 is a small cell adhesion molecule that, among other tyrosine kinase receptors, is able to act on ErbB. It was shown to be a mediator of reverse cardiac remodeling in chronic heart failure [72]. In addition to its effects on ErbB receptors, neuregulin-1 induces paracrine secretion of cytokines in the heart including interleukin-1α and interferon-γ as well as pro-reparative factors (such as angiopoietin-2, brain-derived neurotrophic factor, and crypto-1), which have been demonstrated to contribute to cardiac repair mechanisms [73]. Rats showed exercise-induced upregulation of neuregulin-1 at the mRNA and protein level which was linked with physiological hypertrophy and cardiomyocyte proliferation [74]. Neuregulin-1 signaling can induce expression of transcription factor CAAT/enhancer-binding-protein beta (C/EBPβ) which is known to be involved in exercise-induced cardiac growth and protection in the context of pathological cardiac remodeling [21]. It has been further demonstrated that exercise activates neuregulin-1/ErbB signaling and promotes cardiac repair after myocardial infarction in rats [70] which shows its importance in cardiac regeneration in response to sports.

#### 2.3.3. Nitric Oxide (NO) Signaling

Exercise has been shown to increase circulating catecholamines and consequently the expression of β3 adrenergic receptors (β3-AR) [8,75]. β3-AR stimulation in turn mediates endothelial nitric oxide synthase phosphorylation and increases cardiac NO metabolite levels (nitrite and nitrosothiols) which contribute to cardioprotective effects of exercise in ischemic hearts. Cessation of training in contrast extenuated NO levels and cardioprotective effects [8]. 

Interestingly, Akt signaling again is able to activate eNOS phosphorylating at Ser1176, an activation which has been shown to be critical for early ischemic preconditioning-induced cardioprotection [76]. NO has further been highlighted to inhibit ischemia/reperfusion injury, inflammation, and left ventricular remodeling in the absence of reactive oxygen species. Exercise inhibits ROS generation and promotes bioavailability of NO [77]. NO activates soluble guanylate cyclase, increases cGMP level, and activates PKG. Dysregulation in NO/PKG signaling is known to be involved in heart failure with enhanced calcium handling alterations, fibrosis, titin-based stiffness, pathological cellular hypertrophy, and microvascular dysfunction [78]. PKG in contrast inhibits pressure-induced cardiac remodeling in mice [79].

#### 2.3.4. Other Pathways and Extracellular Vesicles

Physical exercise increases cardiac contractility to supply the raised demand of oxygenated blood. In this context training improves aging-induced downregulation of thyroid hormone receptor signaling mediated transcription of myosin heavy chain (MHC) and sarcoplasmatic reticulum Ca(2+)-ATPase and contributes to an improvement in cardiac function and contractility in aged rat hearts [80]. 

Signaling via extracellular vesicles (exosomes, EVs) has gained interest recently since they act as paracrine signaling mediators. They were found to be secreted by cardiac human progenitor cells containing microRNAs (e.g., miR-210, miR-132) and consequently inhibit cardiac apoptosis and improve cardiac function after acute myocardial infarction [81]. After three weeks of swimming mice showed increased levels of EVs and were more resistant against cardiac ischemia/reperfusion injury and demonstrated antiapoptotic effects by the activation of extracellular signal–regulated kinases 1/2 (ERK1/2) and heat shock protein 27 (HSP27) signaling [82]. Induced pluripotent stem cell (iPSC)-derived EVs seem to be more effective to induce cardiac repair mechanisms (including maintained left ventricular function and vascularization, amelioration of apoptosis, and hypertrophy) compared to iPSCs themselves [83].

## 3. MicroRNAs

MicroRNAs are small noncoding RNA molecules of approximately 22 nucleotides in length modulating gene expression post-transcriptionally by binding to its target messenger RNAs promoting their degradation [84]. Several animal and human studies have shown altered miR levels in cardiac diseases such as hypertrophy, ischemic, and dilated cardiomyopathy, aortic stenosis, or arrhythmias [85,86,87,88,89,90].

As described above cardiac cell regeneration was thought to be a rare event in adult mammalian hearts for a long time but recently different miRNA clusters have been linked to increased cardiomyocyte proliferation and cardiac regeneration whereas others have been related to reduced cardiomyocyte proliferation. Recently, however, animal studies revealed detailed insights in cardiac regeneration processes including cardiomyocyte proliferation in failing hearts [91,92] and inducible regeneration in adult cardiomyocytes [16,17,18]. Renewal of adult cardiomyocytes after acute injury of the heart is therefore present yet insufficient [93]. MicroRNAs are directly linked to control this cardiomyocyte regeneration, renewal, and proliferation. Many of these findings were obtained in exercise animal studies and may further contribute to develop novel innovative therapeutic strategies for patients with failing hearts in the future. Table 1 gives an overview of cardiac microRNAs changes in response to physical activity.

Several microRNAs have been discovered contributing to an improved cardiac repair after distinct cardiac injury [103]. Among those, the miR-17-92 cluster seems to play an important role in regulating cardiac growth, proliferation, hypertrophy, and survival in response to exercise [94]. This microRNA family is known for regulatory influences on cell cycle, apoptosis and proliferation and has been linked to enhanced proliferation and survival of colorectal cancer [104] as well as keratinocyte proliferation and metastasis [105]. Shi et al. confirmed the role of miR-17-3p in cardiomyocyte hypertrophy and proliferation in a rodent model of exercise: Inhibition of miR-17-3p in mice decreased exercise-induced cardiac growth, cardiomyocyte hypertrophy and expression of markers of myocyte proliferation. Furthermore, mice treated with a miR-17-3p agomir received protection against cardiac remodeling after cardiac ischemia. The authors identified that miR-17-3p suppresses TIMP3, an inhibitor of EGFR/JNK/SP-1, a pathway promoting cardiomyocyte proliferation [106], as well as PTEN which antagonizes PI3K/Akt pathway [107], a pathway vital for cardiac hypertrophy [58] as elucidated above. Samples of ventricular cardiomyocytes obtained in patients with dilated cardiomyopathy revealed a downregulation of miR-17-3p, which confirms its important role in cardiac remodeling, growth, and proliferation. 

Exercise-induced activation of PI3K/AKT/mTOR signaling and subsequent left ventricular physiological hypertrophy is not only mediated via disrupted miR-17 levels but also via other miR-clusters as shown in a rat model [63]: Ma et al. found decreased miRNA-124 levels (targeting PI3K) and increased miRNAs-21, -144, and -145 (targeting PTEN and TSC-2), all leading to an induced activation of PI3K/Akt/mTOR signaling in cardiomyocytes. Furthermore swimming exercise and application of recombinant human growth hormone (r-hGH) in rats altered cardiac PI3K/Akt/mTOR signaling and miR-21 and miR-133 expression [96]. Reciprocal repression between miR-133 and calcineurin is involved in regulating cardiac hypertrophy [98]. These findings emphasize the significance of PI3K/Akt signaling in cardiac proliferation and physiological hypertrophy and support the beneficial effects of physical activity.

Importantly, miR-222 was identified as a key regulator of exercise-induced cardiomyocyte growth and proliferation in mice as induced miR-222 expression in cardiomyocytes led to resistance against adverse cardiac remodeling and ventricular dysfunction after ischemia [95]. These effects were mediated via inhibition of p27, Hipk1, and Hmbox1. Inhibition of endogenous miR-199a was shown to contribute to physiological cardiac hypertrophy probably due to the upregulation of PGC1α in treadmill-trained mice [101]. In another promising study treatment with either miR-199a or miR-590 elevated cardiomyocyte proliferation in postnatal mice promoting cell-cycle re-entry, recovered cardiac contractility, and decreased the levels of fibrosis after myocardial infarction [103].

In contrast miR-15 family attenuates heart regeneration through inhibition of postnatal cardiomyocyte proliferation and acute inhibition of miR-15 in adult mice is associated with improved contractile function after ischemic injury [108]. 

Physical activity also leads to altered miR clusters affecting increased cardiac angiogenesis in animal models. Among those miR-15 is involved in controlling angiogenesis and downregulated under hypoxic conditions [109]. Increased miRNA-126 expression is associated with exercise-induced cardiac angiogenesis in response to changes in vascular endothelial growth factor (VEGF) pathway as well as mitogen-activated protein kinase (MAPK) and PI3K/Akt/eNOS pathways in rats [97]. Decline in cardiac microvascularization is a finding often obtained in aging and diabetes mellitus. Exercise training in this context attenuated aging-induced downregulation of VEGF signaling cascades including phosphorylation of Akt and eNOS proteins contributing to an improvement of angiogenesis in old age rats [110]. 

Ventricular stiffness, increase in fibrosis and diastolic dysfunction often accompanies heart failure. MiR-29c was found to be involved in improving ventricular compliance: Exercising rats showed increased miRNA-29c expression correlating with a decrease in collagen I and III expression and improved LV compliance [99]. Exercise-induced release of exosomes from cardiomyocytes containing miR-29b and miR-455 downregulated matrix metalloproteinase 9 (MMP9) resulting in decreased fibrosis and matrix degradation [100].

Swimming-trained mice showed decreased cardiac apoptosis via increased Bcl-2/Bax ratio, an effect mediated via miR-1, miR-30b, and miR-21 [102].

Changes in miRNA expression found in animal studies seem to be at least in part transferable to humans though their significance is still incompletely understood: Increases in circulating miR-126 have been also shown in healthy adults after endurance exercise [111]. miR-1, miR-133a, and miR-206 levels were significantly elevated after exercise and correlated with performance parameters such as maximum oxygen uptake and anaerobic lactate threshold [112]. 

## 4. Metabolic and Mitochondrial Cardiac Changes

Exercising cardiomyocytes predominantly use glucose and fatty acids to generate energy. The systemic usage of substrates is measured via respiratory exchange ratio (RER). A RER of 1.0 represents total usage of carbohydrates while a ratio of 0.7 represents fatty acid usage (values in between represent a mixture). During exercise RER early approaches 1.0 but returns toward 0.7 in extended workouts indicating a shift towards fatty acid oxidation after longer duration [113].

Different metabolic and mitochondrial alterations related to training have been identified. This has high clinical implication for potential therapeutic targets as mitochondrial dysfunction is one of the key findings in heart failure and as exercise is protective for cardiac mitochondria against ischemia/reperfusion injury in mice [114]. Rather inconsistent data about changes in the proportion of glucose and fatty acid oxidation in cardiac cells have been gained in the past years from rodent models. Therefore, exact assignment of substrate utilization in response to chronic exercise remains uncertain [115]. Clear changes have been found in metabolic gene expression and mitochondrial pathways: Expression of genes involved in beta-oxidation of fatty acids and glucose metabolism in the heart differ between exercise-induced physiological cardiac growth and maladaptive pathological cardiac growth. Genes involved in β-oxidation are downregulated in maladaptive cardiac hypertrophy whereas they (including CD36, a fatty acid translocase and scavenger receptor) are found to be upregulated in exercise-induced cardiac hypertrophy [116]. CD36 deficiency is directly linked to insulin resistance and defective fatty acid metabolism in rats [117]. In contrast abnormal myocardial fatty acid uptake via redistribution of CD36 from intracellular stores to the plasma membrane was found in early stages of insulin resistance contributing to cardiac lipotoxicity [118]. The impact of CD36 on cardiac function is not entirely understood. CD36-deficient hearts were found not to be energetically or functionally compromised and were not more vulnerable to ischemia as energy generation through glucose oxidation was able to compensate for the loss of fatty acid-derived energy generation [119].

The transcriptional co-activators PGC-1α and PGC-1β regulate oxidative phosphorylation and fatty acid oxidation gene expression and control number and size of mitochondria. Heart failure is associated with repressed PGC-1α and PGC-1β gene expression [120]. In mice with diabetic cardiomyopathy running prevented cellular apoptosis and fibrosis, improved mitochondrial biogenesis, prevented diabetic cardiomyopathy-associated inhibition of PGC-1α, and activated Akt signaling [121].

Adenosine monophosphate-activated protein kinase is a serine/threonine kinase which participates in regulating cellular energy supply [122]. Long-term activation of AMPK blocks cardiac hypertrophy as well as NFAT, NF-kB, and MAPK signaling and thus preserves cardiac function in pressure-overload rats [123]. AMPK deficiency, on the other hand, exacerbates LV hypertrophy in mice [124]. Swimming-trained rats showed activated AMPK levels with reduced cardiac fibrosis due to inhibition of NADPH oxidase [125]. This finding has been confirmed as decreased AMPK activity by beta-adrenergic activation exacerbated cardiac fibrosis [126]. This is interesting as exercise activates AMPK [127] and consequently might be able to inhibit pathological hypertrophy and cardiac fibrosis.

The intrinsic mitochondrial apoptotic pathway is one of the most important mechanisms of myocyte degeneration in the progression of heart failure [128]. Exercise induces a cardiac mitochondrial phenotype that resists apoptotic stimuli increasing antioxidant enzymes [129]. The Bcl-2 pathway is one of those proapoptotic mitochondrial-mediated pathways and has been demonstrated to be critical in regulating apoptosis in aging patients [130]. Rat hearts showed 12 weeks after training attenuated age-induced elevation in Bax/Bcl-2 ratio and consequently lower apoptotic rates and remodeling [131].

Another key metabolic pathway induced by exercise is the peroxisome proliferator-activated receptors (PPAR)-pathway. PPAR are transcription factors mediating the development of cardiac hypertrophy and regulating fatty acid metabolism [132]. Exercise increases PPAR-alpha levels and decreases consequently inflammatory response including TNF-alpha and NF-kB levels [133]. This is important as PPAR-alpha stimulation downregulates inflammatory molecules and decreases infarct size [134]. Aging processes decrease PPAR-alpha levels, a finding that could be attenuated in swim-trained rat hearts which contributed to an improvement in fatty acid metabolic enzyme activity [135].

## 5. Conclusions

Alterations in cellular pathways including Akt, ErbB, and NO signaling as well as various miR clusters have been linked to cardiac disease such as heart failure. Physical exercise is known to be cardioprotective and can partly compensate cardiac damage as demonstrated in various animal models and patient studies. On a cellular level sports counteracts cardiac disease related alterations in these cellular pathways and is able to improve cardiac function. Insights in cellular cardioprotective pathways obtained from these exercise studies could contribute to the development of novel therapeutic strategies in failing hearts due to toxic, infectious or ischemic injury, or aging in the future.

## Figures and Tables

**Table 1:** Overview of microRNA levels altered in response to physical exercise and their contribution to cardioprotection.

MicroRNA	Cellular Target	Cardiac Function	Animal Model and Exercise Modality	References
**miR-17-3p**	TIMP3, PTEN	Cardiac hypertrophy Myocyte proliferation Cardiac apoptosis	Mice, swimming and wheel exercise	[94]
**miR-222**	P27, Hipk1, Hmbox1	Cell cycle Cardiac apoptosis Cardiac hypertrophy Myocyte proliferation	Mice, swimming and wheel exercise	[95]
**miR-124**	PI3K	Cardiac hypertrophy	Rats, swimming exercise	[63]
**miR-21**	PTEN	Cardiac hypertrophy	Rats, swimming exercise	[63,96]
**miR-144**	PTEN	Cardiac hypertrophy	Rats, swimming exercise	[63]
**miR-145**	TSC	Cardiac hypertrophy	Rats, swimming exercise	[63]
**miR-126**	Spred-1 Raf-1/ERK 1/2 signaling	Cardiac angiogenesis	Rats, swimming exercise	[97]
**miR-133**	Calcineurin PI3K/Akt signaling	Cardiac hypertrophy	Rats, swimming exercise	[96,98]
**miR-29c**	Collagen I und III TGFβ pathway	Left ventricular compliance	Rats, swimming exercise	[99]
**miR-29b**	MMP9	Fibrosis, matrix degradation	Mice, treadmill running	[100]
**miR-455**	MMP9	Fibrosis, matrix degradation	Mice, treadmill running	[100]
**miR-199a**	PGC1α	Cardiac hypertrophy	Mice, treadmill running	[101]
**mi-R1**	Bcl-2	Cardiac apoptosis	Mice, swimming exercise	[102]
**miR-30**	P53, Drp-1	Cardiac apoptosis	Mice, swimming exercise	[102]
**miR-21**	PDCD4	Cardiac apoptosis	Mice, swimming exercise	[102]

Extracellular signal–regulated kinases 1/2 (ERK1/2).
